# Smart Gateway for Healthcare Networks Based on Beam Steering Technology

**DOI:** 10.3390/s23062959

**Published:** 2023-03-09

**Authors:** Kazuhiro Honda, Kosuke Takakura, Yuki Otsubo

**Affiliations:** Graduate School of Engineering, University of Toyama, 3190 Gofuku, Toyama 930-8555, Japan

**Keywords:** circular array, interferometric angle-of-arrival (AOA) estimation, beam steering, over-the-air (OTA) testing, beam propagation experiment

## Abstract

To ensure high-reliability communication in healthcare networks, this paper presents a smart gateway system that includes an angle-of-arrival (AOA) estimation and a beam steering function for a small circular antenna array. To form a beam toward healthcare sensors, the proposed antenna estimates the direction of the sensors utilizing the radio-frequency-based interferometric monopulse technique. The fabricated antenna was assessed based on the measurements of complex directivity and the over-the-air (OTA) testing in Rice propagation environments using a two-dimensional fading emulator. The measurement results reveal that the accuracy of the AOA estimation agrees well with that of the analytical data obtained through the Monte Carlo simulation. This antenna is embedded with a beam steering function employing phased array technology, which can form a beam spaced at 45° intervals. The ability of full-azimuth beam steering with regard to the proposed antenna was evaluated by beam propagation experiments using a human phantom in an indoor environment. The received signal of the proposed antenna with beam steering increases more than that of a conventional dipole antenna, confirming that the developed antenna has great potential of achieving high-reliability communication in a healthcare network.

## 1. Introduction

Nowadays, the development of artificial intelligence (AI), big data (BD) analysis, and wireless communication technology is essential to address global social problems, such as industrial innovation, energy saving, medical care, and education inequality [1,2,3,4]. To solve these issues, one of the most important technologies is realizing a world where everything is connected to the internet, or the so-called ‘internet of things’ (IoT). Several studies realizing a smart society utilizing IoT have been reported [5,6,7,8].

**Figure 1:**
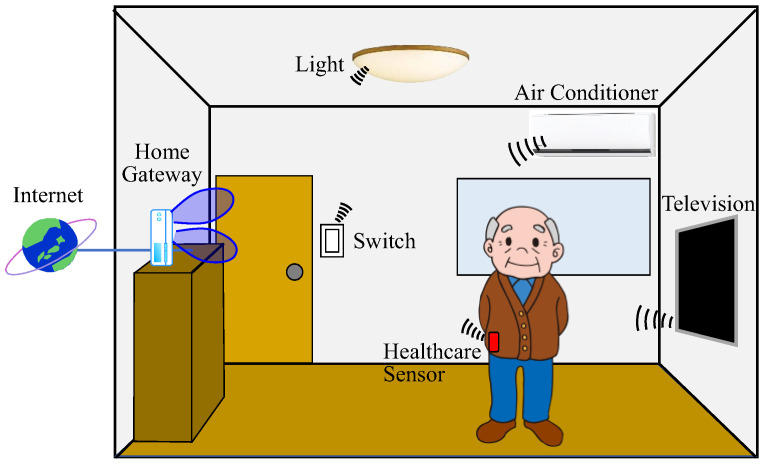
Conceptual illustration of a smart home.

Figure 1 is a conceptual illustration of a smart home, which can help realize a sustainable society by controlling home appliances to save energy or use sensors attached to the human body to help a person stay healthy. IoT devices are connected to the internet through not only fifth-generation mobile communication (5G) [9], for which massive machine-type communication is rather standardized, but they also use the IEEE 802.11 standard [10], commonly known as WiFi, Bluetooth [11], RFID [12], and low-power wide-area networks (LPWAN), such as SigFox [13], LoRa [14], and NB-IoT [15]. With regard to most smart home applications, many IoT devices probably will be connected using WiFi.

The data size transmitted from IoT devices is small, so high-capacity communication such as 5G is unnecessary. However, if the purpose of innovating sensor devices is to monitor the condition of the human body or attached instruments, it is necessary to continuously communicate with sensing data. A method to achieve high-reliability communication is to form a beam in the direction of the communication target at the receiver end. Consequently, a significant improvement in received signal level is expected compared to the conventional method using an antenna with omni-directivity. A receiving device must then have two distinct features: localization and beamforming. In particular, since healthcare sensors are usually mounted on the human body, the sensors’ position is expected to change drastically during walking or other motion, so tracking these sensors is a very important issue.

The global positioning system (GPS) is known as a high-accuracy localization method for outdoors in situations where a receiving device can be confirmed by multiple GPS satellites, i.e., in a multiple line-of-sight (LOS) environment. If the sensor device can transmit the position information obtained by GPS to the receiver, the gateway does not need to locate these sensors. However, most indoor situations are non-line-of-sight (NLOS) propagation and, therefore, GPS satellites cannot see them directly. Many sensor devices are used in buildings, factories, and houses, so there has been great interest in indoor localization methods that have a high estimation accuracy [16].

A simple localization technique is the received signal strength indication (RSSI) method. This method guesses the location using triangulation based on multi-distance calculation of the path losses between the sensor and the access points installed indoors. Similar methods exist based on the time required for communication between the sensor and the access points. Because the received signal fluctuates due to the influence of the multipath, mitigation methods to reduce the degraded estimation accuracy are being investigated [17,18]. Consequently, these estimation methods require multiple access points, and, moreover, the access points themselves cannot identify the location of the sensor device. On the other hand, a method using channel state information (CSI), which is based on the radio propagation characteristics between terminals, has also been reported [19,20]. This method localizes the position based on the amplitude and phase difference between multiple transmitting antennas and multiple receiving antennas. The localization method based on CSI achieves a high accuracy estimation, but it requires specific hardware and software.

One of the smart antenna systems that has the required attributes of localization and beamforming is the electric-steering parasitic array radiator (ESPER) antenna [21]. This antenna consists of an active element at the center and several parasitic elements arranged in a circle. The beam is created by terminating the parasitic elements with optimum load impedances depending on the desired direction. The angle of the incoming wave is estimated by using signal processing techniques, such as the multiple signal classification (MUSIC) algorithm and the estimation of signal parameters via the rotational invariance technique (ESPRIT) algorithm [22,23]. Stable communication is realized by generating a beam in the direction estimated. However, it is known that the estimation accuracy of these methods depends on the signal-to-noise ratio (SNR), resulting in a difficulty with estimating in hostile environments.

In our previous study, to realize the position for ground-based vehicle applications, an angle-of-arrival (AOA) antenna system that utilizes the radio-frequency (RF)-based interferometric monopulse technique was proposed [24,25]. The proposed AOA antenna has a high degree of accuracy regardless of the K-factor, which expresses the ratio of the direct wave power to the scattered wave power in a LOS propagation environment. However, the proposed antenna in this case was composed of nine elements to achieve a full-azimuth beamforming multiple-input multiple-output (MIMO) function. For a small antenna with a reduced number of elements that does not require high-capacity communication, an AOA estimation of the incoming wave and beamforming in the desired direction has not been considered.

This paper presents a smart gateway system that includes the AOA estimation and the full-azimuth beam steering functions for a small circular antenna array. The proposed antenna was redesigned to increase the radiation gain and to improve the estimation accuracy of the incoming wave based on previous observations. The fabricated antenna was assessed for its estimation accuracy of incoming wave using over-the-air (OTA) testing in Rice propagation environments with a two-dimensional fading emulator. Moreover, the ability of the beam steering in the proposed antenna was evaluated by propagation experiments using a human phantom model in an indoor environment.

The remainder of this paper is organized as follows: in Section 2 the estimation method and beamforming for the proposed antenna are described; Section 3 shows the directivity measurements made in an anechoic chamber; Section 4 shows the verification of the estimation accuracy using an OTA apparatus; Section 5 shows the experimental results of the beam steering function in an indoor environment; and Section 6 concludes the paper.

## 2. Smart Gateway Antenna

**Figure 2:**
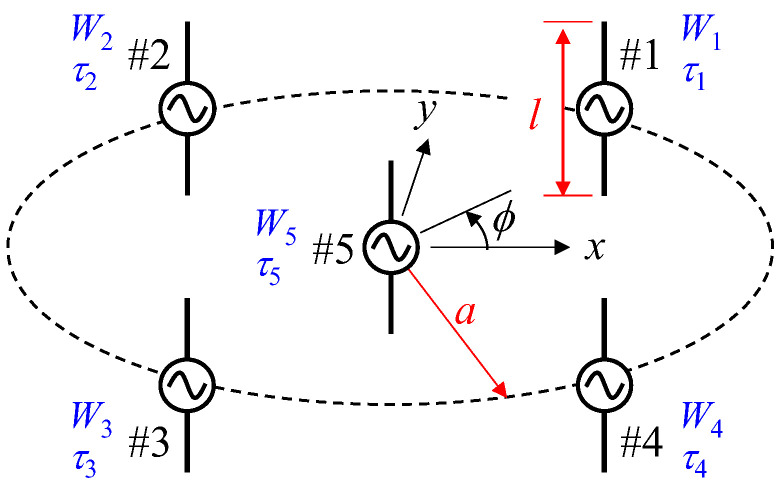
Configuration of the smart gateway antenna.

Figure 2 shows the configuration of the proposed antenna. This antenna is composed of five active elements, four of which (#1–#4) are arranged at equal angular intervals of 90° on a circle of radius *a*, with the final element (#5) located at the center of the circle. The arrangement of the elements is the same as the ESPAR antenna, except that all elements are excited. By controlling the weight *w* and phase shift *τ* of the excitation, it is possible to form the directivity of two functions: AOA estimation and beam steering.

The polarization characteristics of the radio wave radiated from the sensor, which is attached to the human body, will change significantly depending on the mounting orientation of the sensor and human motion, regardless of the radiation characteristics of the sensor antenna. We had previously reported a weighted-polarization wearable MIMO antenna, which can have controlled polarization characteristics by considering the variation in the cross-polarization power ratio of the incoming wave and the antenna inclination angle, which depends on the human motion [26]. However, the development of this sensor is not considered in this study. Although it is desirable for the gateway antenna to receive both polarizations, this study deals only with the vertical polarization. Therefore, all the antenna elements in Figure 2 are for a dipole antenna perpendicular to the ground. *l* is the length of an element. The radius *a* and the length *l* of the proposed smart gateway antenna were redesigned to increase the radiation gain and improve the estimation accuracy of the incoming wave based on the results of a previous antenna that is composed of nine elements [24,25]. The operating frequency is 2 GHz.

### 2.1. Interferometric AOA Estimation Method

The proposed antenna estimates the angle of the incoming wave using a radio-frequency (RF)-based interferometric monopulse technique. The Butler matrix, which is used to give an appropriate phase delay to the received signals [27], has the drawback of being a bulky microwave component, which leads to hardware complexity. The AOA method proposed in this paper employs an RF-based interferometric monopulse technique without a Butler matrix, which reduces hardware complexity.

Here, we explain the AOA estimation method. When a radio wave originates from the *ϕ* direction, as measured from the *x*-axis, the voltage induced on the *i*-th element located on the circle with radius *a* can be calculated using the following equation:

(1) $$V_{i}=e^{j\frac{2\pi }{\lambda }a\cos\left(\frac{2i-1}{4}\pi -\phi \right)}$$

where all the elements are assumed to be isotropic antennas, and *λ* denotes the wavelength of the received signal.

The voltage *V_i_* is multiplied by the phase shift *τ_i_*, depending on the element arrangement angle, as defined by the following equation:

(2) $$\tau_{i}=-\frac{2i-1}{4}\pi$$

when all the weights are set to one, the summation of the total voltage induced on the four elements can therefore be written as follows:

(3) $$E_{\Delta }=\sum_{i=1}^{4}e^{j\left\{\frac{2\pi }{\lambda }a\cos\left(\frac{2i-1}{4}\pi -\phi \right)\;-\;\frac{2i-1}{4}\pi \right\}}$$

Now, we consider the case where the number of elements is infinite and *E*_∆_ converges to the Bessel function, as expressed by the following equation:

(4) $$E_{\Delta }=e^{-j\left(\phi -\frac{\pi }{2}\right)}\cdot 2\pi \;J_{1}\left(\frac{2\pi }{\lambda }a\right)$$

The received signal at the fifth element when *w*_5_ = 1 and *τ*_5_ = 0 is represented by *E*_Ω_. Furthermore, the phases of *E*_∆_ and *E*_Ω_ are denoted by ∠*E*_∆_ and ∠*E*_Ω_, respectively. The phase difference is calculated as follows:

(5) $$\phi_{m}=∠E_{\Delta }-∠E_{\Omega }$$

The phase difference expressed by Equation (5) is approximately proportional to *ϕ* (the actual direction of an incoming wave) because ∠*E*_Ω_ is constant regardless of the angle of the incoming wave. The effect of mutual couplings is not adequately considered in the theoretical formula. In fact, the electromagnetic mutual coupling between the elements occurs, so the amplitude and phase of the directivity will be fluctuating, resulting in an estimation angle error of the incoming wave.

**Figure 3:**
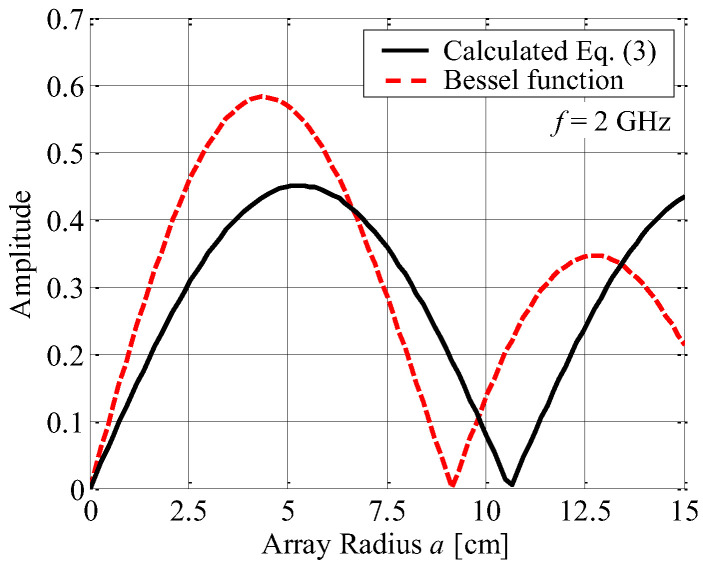
Summation of the whole voltage induced on the four elements.

Figure 3 shows the summation of the whole voltage denoted by Equation (3) as a function of the array radius *a*, together with the Bessel function drawn as the red broken curve. The magnitude of *E*_∆_ reaches a maximum value when the radius is 5.3 cm. This configuration of a narrow element spacing may lead to a significant gain reduction in the antenna due to the strong electromagnetic mutual coupling.

**Figure 4:**
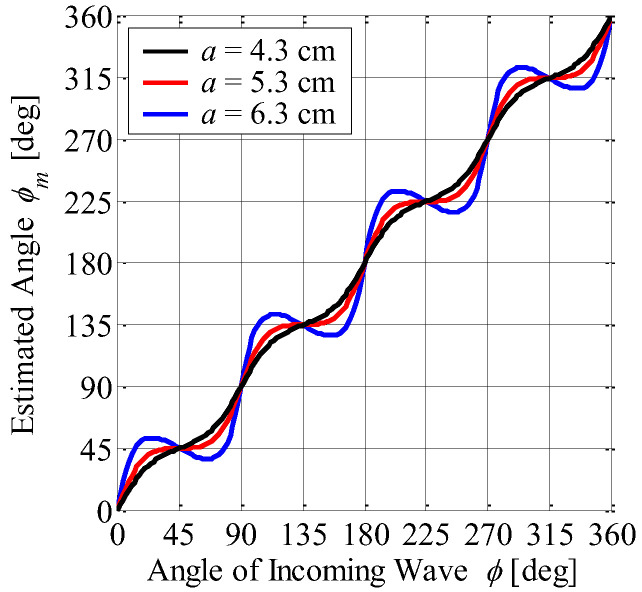
Estimation angle vs. angle of incoming wave.

Figure 4 shows the estimated angle *ϕ_m_* defined by Equation (5) as a function of the actual azimuth angle *ϕ* with the array radius as a parameter. It can be seen from Figure 4 that the estimated angle is approximately proportional to the actual azimuth angle. However, the error of the estimation angle of the incoming wave increases with increasing array radius, indicating a tradeoff relationship between the radiation gain and the accuracy of the estimation angle.

In general, a narrow element spacing may lead to a significant directivity fluctuation due to strong electromagnetic mutual coupling. To fulfill high performance in a small gateway antenna system, the proposed antenna needs to be redesigned to increase the radiation gain and to improve the estimation accuracy of the angle of the incoming wave. The directivity of the AOA estimation function with regard to the proposed antenna is calculated using the method of moments, and the effect of electromagnetic mutual coupling on the characteristics of the proposed antenna has been investigated. The dipole antenna with length *l* is used for the five array elements shown in Figure 2. All the dipoles are terminated at 50 Ω. It should be noted that, although the proposed smart gateway antenna is a receiving antenna, the analysis is conducted with a transmitting antenna on the basis of the reciprocal theorem throughout this paper.

**Figure 5:**
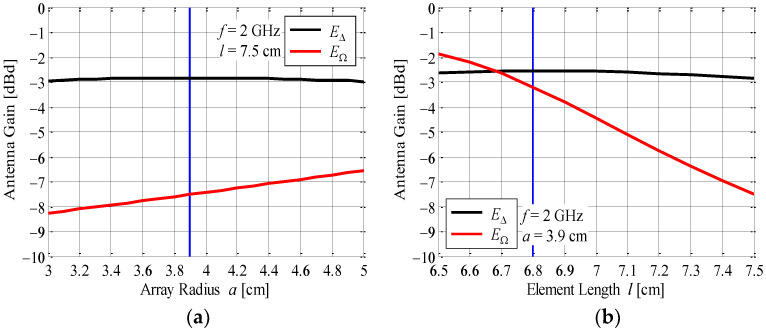
Radiation gain of the AOA estimation function in the proposed antenna: (**a**) array radius and (**b**) element length.

Figure 5a shows the radiation gain in *E*_∆_ and *E*_Ω_ at *ϕ* = 0° as a function of the array radius. The radiation gain takes into account the mismatch loss in order to consider the effects of the electromagnetic mutual coupling. For information, the array radius of a previous AOA antenna with eight elements on the circle is 4.9 cm [24,25]. The black and red curves indicate the radiation gain in *E*_∆_ and *E*_Ω_, respectively. The element length is set to 7.5 cm, i.e., half-wavelength.

As shown in Figure 5a, the radiation gain in *E*_Ω_ increases with increasing array radius. On the other hand, that of *E*_∆_ is almost constant but is largest at *a* = 3.9 cm, that is, at 0.26 λ, it is roughly equivalent in size to the conventional ESPER antenna. Hence, we determined the array radius to be 3.9 cm because the accuracy of the estimation angle of the incoming wave may decrease with increasing array radius, as shown in Figure 4. Consequently, the distance between the elements, which are arranged at equal angular intervals on a circle, is approximately 5.5 cm, that is, at 0.37 *λ*.

Figure 5b shows the radiation gain in *E*_∆_ and *E*_Ω_ at *ϕ* = 0° as a function of the element length. The radiation gain takes into account the mismatch loss in order to consider the effects of electromagnetic mutual coupling. The black and red curves indicate the radiation gain in *E*_∆_ and *E*_Ω_, respectively. The array radius is set to 3.9 cm.

As shown in Figure 5b, the radiation gain in *E*_Ω_ decreases with increasing element length. Similar to Figure 5a, that of *E*_∆_ is almost constant but is largest at *l* = 6.8 cm, that is, at 0.45 *λ*. Hence, we determined the element length to be 6.8 cm because a shorter element length deviates from the operating principle of a half-wavelength dipole antenna.

**Figure 6:**
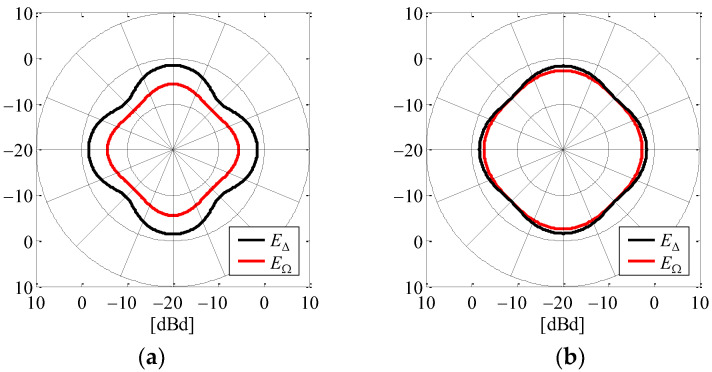
Amplitude radiation pattern of the AOA estimation function: (**a**) *a* = 4.9 cm and *l* = 7.5 cm; (**b**) *a* = 3.9 cm and *l* = 6.8 cm.

Figure 6 shows the calculated amplitude radiation pattern of *E*_∆_ and *E*_Ω_ defined in the *xy*-plane. Figure 6a is the case of *a* = 4.9 cm and *l* = 7.5 cm as the configuration before the redesign, in which the number of elements has been reduced from the conventional AOA antenna [24,25]. Figure 6b shows the case of *a* = 3.9 cm and *l* = 6.8 cm as the configuration after the redesign. The black curve indicates the radiation pattern of *E*_∆_, whereas the red curve is the radiation pattern of *E*_Ω_.

It can be seen from Figure 6 that the difference between the maximum and minimum gains of the directivity after the redesign is smaller than that before the redesign for both *E*_∆_ and *E*_Ω_, but especially for *E*_∆_, thereby enabling a more omnidirectional radiation pattern. Moreover, the radiation gain in *E*_Ω_ after the redesign is greater than that before the redesign. Hence, the received level of *E*_Ω_ is expected to improve by about 4 dB.

**Figure 7:**
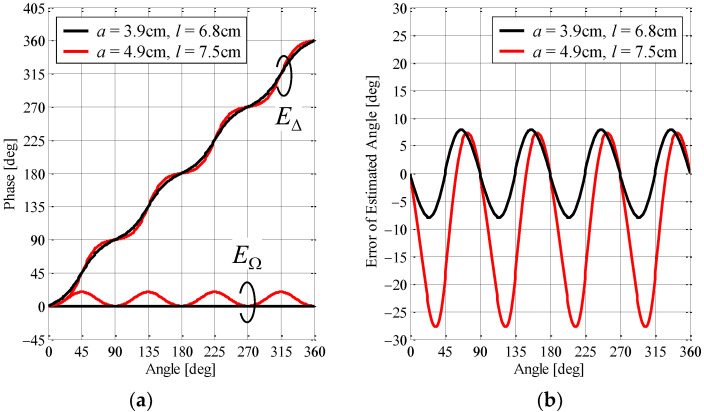
Analytical results of the angle estimated by the proposed antenna: (**a**) phase characteristics and (**b**) estimation error.

To verify the accuracy of the estimation angle of the incoming wave, the radio-frequency (RF)-based interferometric monopulse technique was performed using the calculated complex radiation pattern. Figure 7a shows the phase characteristics of *E*_∆_ and *E*_Ω_ as a function of the azimuth angle. The black and red lines indicate the configuration after and before the redesign, respectively.

From the analytical results presented in Figure 7a, the severe fluctuations that existed before the redesign have disappeared in the phase characteristics of *E*_∆_, indicating that this is suitable as a reference angle. However, the phase characteristics of *E*_∆_ after the redesign also have oscillations, but they are smaller than those before the redesign. The reason is that the amplitude radiation pattern of *E*_∆_ approaches an omnidirectional form, as shown in Figure 6. The phase characteristics of *E*_∆_ seem to be at the limit because the radiation gain does not change much regardless of the array radius and element length, as shown in Figure 5. The improvement in the phase characteristics of *E*_∆_ will be a subject for future study.

Figure 7b shows the error of the estimated angle of the incoming wave as a function of the actual angle. The black and red lines indicate the configuration after and before the redesign, respectively.

The analytical results reveal that the estimation error before the redesign is large due to the synergistic effect of the fluctuations in *E*_∆_ and *E*_Ω_. On the other hand, the estimation error after the redesign is suppressed compared to that before the redesign, depending on the immovable reference characteristics, indicating that the proposed antenna can autonomously estimate the direction of the sensor device.

### 2.2. Mean IQ-Value Method in Rice Propagation Environment

In an indoor situation, even when the smart gateway antenna can be visible to the sensor device, i.e., an LOS environment, there is not only a strong direct wave coming from the sensor device attached to the human body, but there are also reflected and diffracted waves from the surrounding objects. This situation is known as a Rice propagation environment. Therefore, it is necessary to verify the AOA estimation accuracy in a multipath propagation environment, and a method to realize AOA estimation in Rician channels is described below.

**Figure 8:**
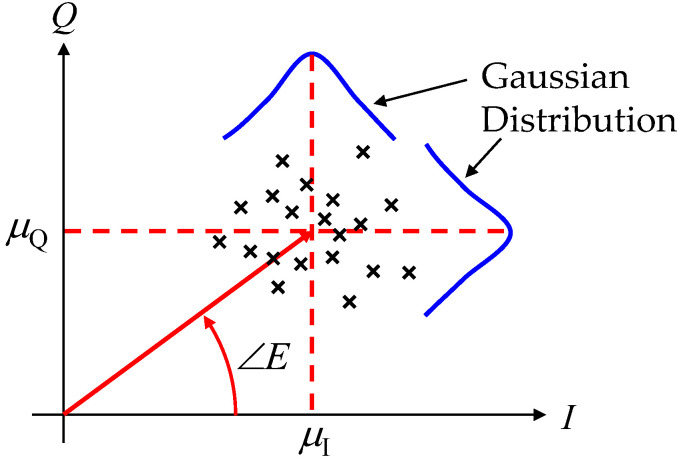
Mean IQ-value method for estimating the angle of incoming waves in a Rice propagation environment.

There is a great deal of difficulty in making interferometric AOA estimations when the direct wave is small in magnitude compared to the summation of the reflected waves, resulting in a small K-factor. This is due to the occurrence of significant phase perturbations caused by scattered reflected and diffracted waves. In [28,29,30], incoming waves are assumed to be plane waves, but multipath effects are not treated. In this paper, we propose a novel algorithm, called the mean IQ-value method (MIQ method), to extract the true angular information of incoming waves in severe Rician fading channels [24]. Figure 8 illustrates the schematic diagram that explains the MIQ method for estimating the angle of incoming waves. In Figure 8, the black symbols signify the complex samples of Rice fading signals. As shown in Figure 8, the random multipath scattered-field components are distributed around a coherent LOS component in the Rician fading channel.

The magnitudes of the multipath scattered fields have the statistical characteristics of the Rayleigh response, meaning that the real and imaginary parts (in-phase and quadrature-phase: named the IQ components) of the multipath components have normal or Gaussian distributions. The probability density functions (PDFs) of the IQ-multipath components have symmetrical bell-shaped curves, as illustrated in Figure 8. This symmetrical nature of the PDFs means that although there are considerable fluctuations for each sample point, their average values (denoted by *μ*_I_ and *μ*_Q_ in Figure 8 for in-phase and quadrature-phase components, respectively) maintain a certain constant value. Based on this theoretical consideration, we propose that the most probable angle of incoming waves can be extracted from the following equation:

(6) $$∠E=\tan^{-1}\frac{\mu_{Q}}{\mu_{I}}$$

**Figure 9:**
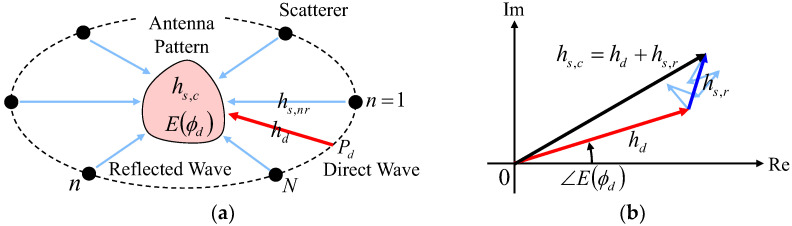
Channel model dependent multipath characterization: (**a**) Rice channel model and (**b**) vector diagram.

To analyze the estimation accuracy of the proposed antenna in a Rician propagation environment, we describe an analytical investigation of channel modeling considering the Rice factor, which is the ratio of the direct wave power and the average scattered wave power, dependent multipath characterization. Figure 9a shows the Rice channel model, whereas Figure 9b is the vector diagram of signals in the complex plane. A Monte Carlo simulation was conducted using this model [31]. Hereafter, we consider the proposed antenna to be located at the center of the circle where the scatterer is placed, as depicted by the broken line in Figure 9a.

The average power of multipath scattered waves is obtained as follows:

(7) $$P_{r}=\frac{1}{S}\sum_{s=1}^{S}{\left|h_{s,r}\right|}^{2}$$

where *S* denotes the number of snapshots for the Monte Carlo simulation. *h_s,r_* (illustrated as the blue arrow in Figure 9b) denotes the channel response of scattered fields and is represented by the following equation:

(8) $$h_{s,r}=\sum_{n=1}^{N}h_{n}\exp\left\{j\frac{2\pi d_{s}}{\lambda }\cos\left(\phi_{n}-\phi_{v}\right)\right\}=\sum_{n=1}^{N}h_{s,nr}$$

where *N* denotes the number of scatterers; *h_n_* indicates the amplitude of the scattered wave; *d_s_* is the moving distance of the antenna; *ϕ_n_* signifies the arrangement angle of the scatterers; and *ϕ_v_* is the moving direction of the antenna.

Then, the channel response of a direct wave *h_d_* (illustrated as the red arrow in Figure 9b) can be expressed as follows:

(9) $$h_{d}=\sqrt{2KP_{r}}\frac{E\left(\phi_{d}\right)}{\left|E\left(\phi_{d}\right)\right|}$$

where *K* is defined as the ratio of *P_d_* to *P_r_*, which is usually known as the Rice factor or K-factor. *E*(*ϕ_d_*) denotes the complex electric field directivity of the AOA estimation function in the proposed antenna (*E*_∆_ or *E*_Ω_). As shown in Figure 9b, the signal response *h_s,c_* (illustrated as the black arrow in Figure 9b) is expressed as follows:

(10) $$\begin{array}{l} h_{s,c}=h_{d}+h_{s,r}\\ \;\;\;=\sqrt{2KP_{r}}\frac{E\left(\phi_{d}\right)}{\left|E\left(\phi_{d}\right)\right|}+\sum_{n=1}^{N}\left(h_{n}\exp\left\{j\frac{2\pi d_{s}}{\lambda }\cos\left(\phi_{n}-\phi_{v}\right)\right\}\right) \end{array}$$

**Figure 10:**
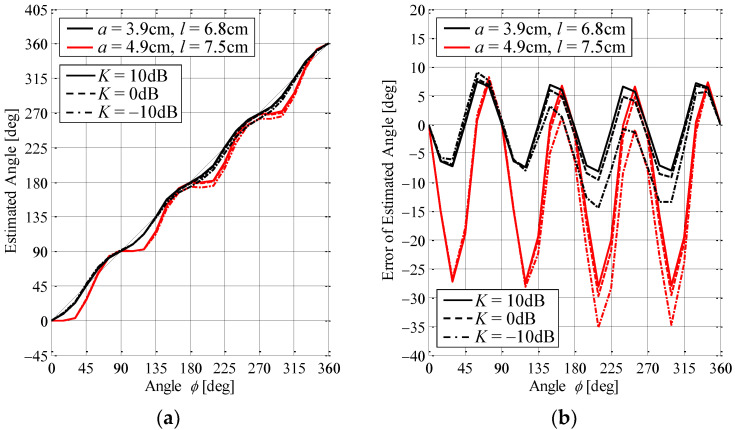
Estimation accuracy of the proposed antenna through Monte Carlo simulation: (**a**) estimated angle and (**b**) error of estimated angle.

Figure 10a shows the results of the estimation of the AOA based on the cooperative operation of the MIQ method with the K-factor as a parameter, whereas Figure 10b is the estimation error of that method. The solid lines, the broken lines, and the chain lines show the case of *K* = 10 dB, 0 dB, and −10 dB, respectively. The black and red lines indicate the configuration after and before the redesign, respectively. In the Monte Carlo simulation, the cross-polarization power ratio (XPR) is set to 50 dB, which is equivalent to a vertically polarized propagation environment. The number of snapshots is 5000. The initial phase of the scatterers is generated by a random number.

It can be seen from Figure 10a that the estimated angle of the proposed antenna after the redesign, as given by Equation (6), agrees well with the actual angle of the incoming wave, as denoted by the thin black line, regardless of the K-factor. In contrast, the estimation angle before the redesign observes a sinusoidal variation.

Furthermore, Figure 10b shows that the estimation error of the proposed antenna before the redesign is from about −30° to 10° regardless of the K-factor. However, after the redesign, this is less than ±10° even in the severe case of *K* = −10 dB, where there are large signal fluctuations due to highly scattered fields, as if it is a free-space environment. For the beamforming function of the proposed antenna described in the next section, since the beams for high-reliability communication are formed toward the estimated angle, the large estimated error causes a discrepancy between the beam direction and the actual incoming wave angle, resulting in a degradation of the received power. If the estimated error is within ±10°, the radiation gain in the direction of the actual incoming wave is expected to be within the half-width of the formed beam. We can, therefore, conclude that the ability of the AOA estimation method in the proposed antenna after the redesign, in cooperation with the MIQ method, gives a remarkably high accuracy even when the LOS component is extremely small.

### 2.3. Beam Steering Function

In 5G, which is currently in practical use around the world, beamforming technology is utilized to reduce the impact on communication performance caused by increased propagation loss from the use of millimeter-wave band. Controlling the beam in the direction of the sensor leads to increased received signal levels, resulting in high-reliable communications.

The beamforming in the ESPER antenna, which element arrangement is similar to the proposed antenna, is achieved by exciting only one element and terminating the other elements with load impedances in order to act as a radiator or reflector, similar to a Yagi-Uda array antenna. Unlike the ESPER antenna, the elements on the circle of the proposed antenna are employed as excitation elements during the AOA estimation. Therefore, those are also used as excitation elements to create a beam toward the direction of the sensor.

With regard to a phased array antenna, the received signals of each element are combined with a phase difference to achieve in-phase excitation in a desired direction, which yields a strong radiation gain. When isotropic point sources are used in the proposed antenna, the weight functions can be uniquely determined by the geometric phase difference between the antennas. However, when dipole antennas are applied, the directivity of each element changes from omnidirectional due to the electromagnetic mutual coupling, so it is necessary to find the optimal weight functions, which depend on the distorted directivity.

The weight functions are determined in such a way that the gain in the communication direction of the combined radiation pattern yields a maximum value [31]. All elements are phase-shifted by *τ_i_* to be in the in-phase state at the angle of the incoming wave in consideration of the electromagnetic mutual coupling. Furthermore, the amplitude of the excitation voltage of each element, *w_i_*, is controlled according to the radiation gain in each element at the angle of the incoming wave.

As the four elements form a circular arrangement with 90° intervals, the beam can be rotated every 90° in the azimuth plane by applying the same weight function. Moreover, by taking advantage of the symmetry feature of the circular array, the beam direction should be considered in the range of 0 to 45°. Table 1 summarizes the optimal weight functions in all elements of the proposed antenna for creating the beam toward the azimuth angle *ϕ*. In Table 1, the weight *w_i_* is normalized by the maximum value, and the phase shift *τ_i_* is based on the element with the greatest weight. The directivity of each element, which is utilized in the search for the optimal weight function, is calculated using the method of moments. All dipole antennas are terminated with 50 Ω.

**Figure 11:**
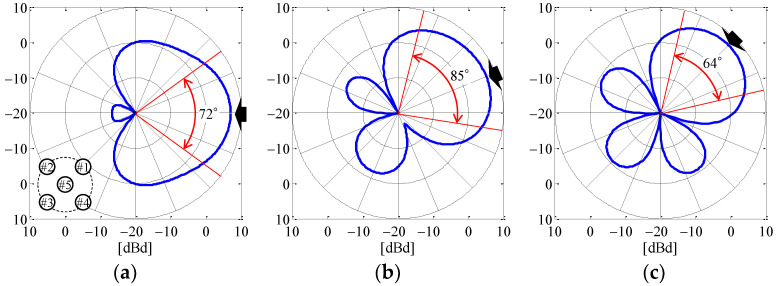
Radiation beam based on Table 1 in the *xy*-plane: (**a**) *ϕ* = 0°; (**b**) *ϕ* = 22.5°; and (**c**) *ϕ* = 45°.

Figure 11 shows the *θ*-polarized radiation beam based on Table 1 in the *xy*-plane as a function of the angle of the incoming wave. The black arrows in the figure indicate the angle of the incoming wave. The red arrows are the full width at half maximum of the radiation gain.

As can be seen from Figure 11, the beam can be formed in the desired direction and produce a radiation gain of about 7 dBd (9 dBi) at the angle of the incoming wave, which results in a large SNR. This maximum radiation gain is greater than that reported in the literature [32]. Moreover, a full width at half maximum of 60° or more can be observed, so a high radiation gain can be expected in all directions by beam steering according to the estimated angle of the incoming wave. However, unnecessary high sidelobes, such as those seen in Figure 11c, are observed, and these are expected to have a negative impact on the reception of interfering waves. Sidelobe reduction is one of the key requirements of beamforming. Many reports on this subject have focused on beamforming toward the direction orthogonal to the array, and there have been few studies in the direction parallel to the array. Although studies investigating multiple elements on a circle or unequally spaced arrangements have been reported [33,34], countermeasures for a circular phased array antenna with few elements that are equally arranged, such as the proposed antenna, have not been reported so far. Sidelobe reduction will be a subject for our future studies. Based on the above analysis, the proposed antenna can simultaneously achieve a high estimation accuracy for the incoming wave and beam steering with a high radiation gain in the full-azimuth angle.

## 3. Directivity Measurements

To confirm the validity of the proposed antenna, we fabricated the proposed design and performed basic experiments using it. In this section, two types of directivities are presented: One is *E*_∆_ with an AOA network and *E*_Ω_, which are employed to estimate the angle of the incoming wave, as described in Section 2.1. The other is beam steering for enhancing the reliability of communications.

### 3.1. Interferometric AOA Estimation

**Figure 12:**
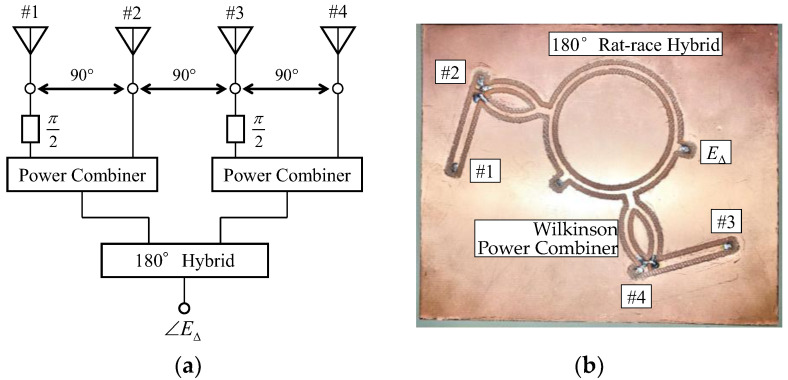
Small-sized AOA network: (**a**) schematic diagram and (**b**) fabricated AOA network.

To implement the weight functions defined by Equation (2), we introduce the use of a simple AOA network. Figure 12 shows the network that generates the received signal *E*_∆_ utilized for the AOA estimation. Figure 12a illustrates a schematic diagram of an AOA network, whereas Figure 12b depicts a fabricated microwave circuit using a double-sided printed circuit board (an FR4-based PCB) with a thickness of 1 mm and a relative permittivity of 4.2.

The phase of the received signals for each element is delayed in accordance with Equation (2). A 180°, a rat-race hybrid allows a 180° phase reversal to be involved in the two signals, and the 90° phase difference for the two signals synthesized in the two Wilkinson power combiners is introduced by using differing lengths of microstrip transmission lines, enabling a small-sized AOA network to be realized.

**Figure 13:**
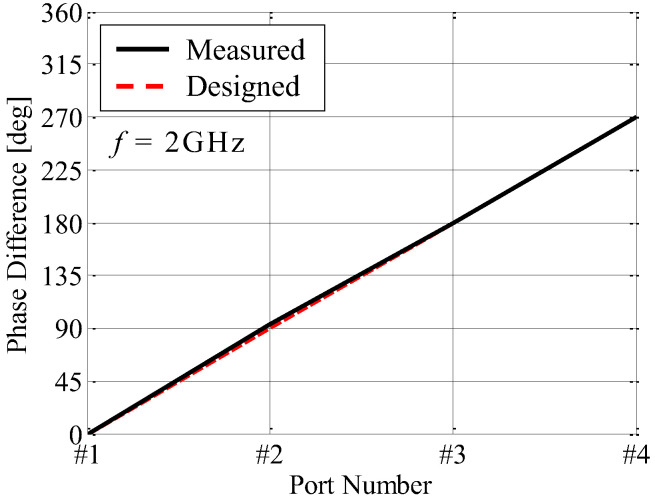
Phase characteristics of the fabricated AOA network.

Figure 13 shows the phase characteristics of the fabricated AOA network. The phase difference between Port #*i* (*i* = 1, 2, …, 4) and output port (*E*_∆_) is measured at 2 GHz using a vector network analyzer. In Figure 13, the phase difference between Port #1 and the output port is used as the reference.

As shown in Figure 13, the measured phase increases by 90° intervals with an increase in the element number. Furthermore, the difference between the measured and designed phases is 1.6° on average. These facts indicate that the fabricated AOA network works properly to achieve the operation of the weight function given by Equation (2).

An experimental verification of the AOA estimation function in the proposed antenna was conducted in an anechoic chamber. The fabricated antenna is composed of four dipole antennas (#1–#4), which are mounted on a circle with a radius *a* = 3.9 cm, and another dipole antenna (#5) is located at the center of the circle. The length of all dipole antennas is 6.8 cm. A photograph of the fabricated antenna will be presented later.

**Figure 14:**
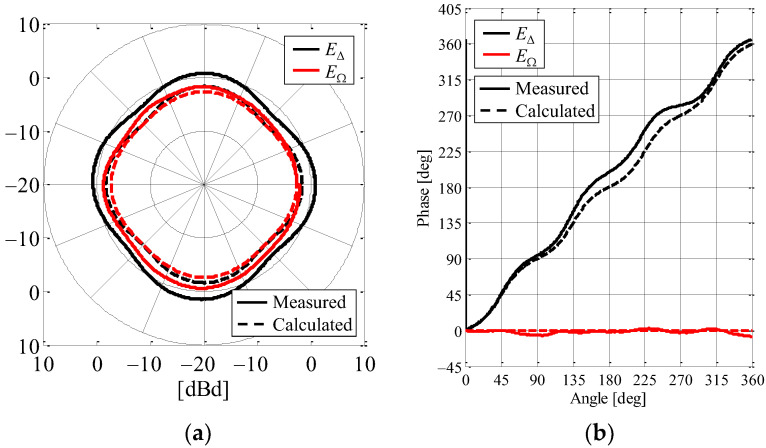
Measured complex directivity for the AOA estimation function: (**a**) amplitude and (**b**) phase.

Figure 14 shows the complex radiation patterns of *E*_∆_ and *E*_Ω_ defined in the *xy*-plane. Figure 14a,b show the amplitude and the phase characteristics, respectively. The black curves depict the vertical polarization component of *E*_∆_, whereas the red curves are that of *E*_Ω_. The solid and broken curves indicate the measured and calculated results, respectively.

As shown in Figure 14a, the measured radiation patterns of *E*_∆_ and *E*_Ω_ agree well with the calculated patterns obtained through the method of moments and have an omnidirectional pattern overall. The maximum radiation gain is approximately 0 dBd, which is equivalent to that of a half-wavelength dipole antenna. Hence, the fabricated antenna can receive the signal radiated from the sensor device during the AOA estimation.

In Figure 14b, the measured phase characteristic of *E*_Ω_ coincides with the calculated value and remains constant regardless of the azimuth angle, indicating that this works as the reference angle for the AOA estimation. On the other hand, there is a little difference between the measured phase characteristic of *E*_∆_ and the calculated value at the azimuth angles from 135° to 270°. The reason for this is the electromagnetic distortion arising from the coaxial cables connected between the dipole antennas and the AOA network circuit. However, the two phases’ characteristics are generally consistent, indicating that the angle of the incoming wave can be estimated by the proposed antenna.

### 3.2. Beam Steering Function

In the AOA estimation function of the proposed antenna, since the weights for *E*_∆_, which indicate the mixing ratio of each element, are the same value, a generalized equal power combiner is sufficient. However, for the beam steering function, the weight *w_i_* of each element is different, as shown in Table 1, so an unequal power combiner is needed to create the beam. In this paper, we unavoidably conducted a preliminary experiment for evaluating the radiation pattern of the beam steering function by using an equal power combiner. The development of a five-way unequal power combiner circuit, such as a two-way combiner [35], will be a subject for our future studies.

**Figure 15:**
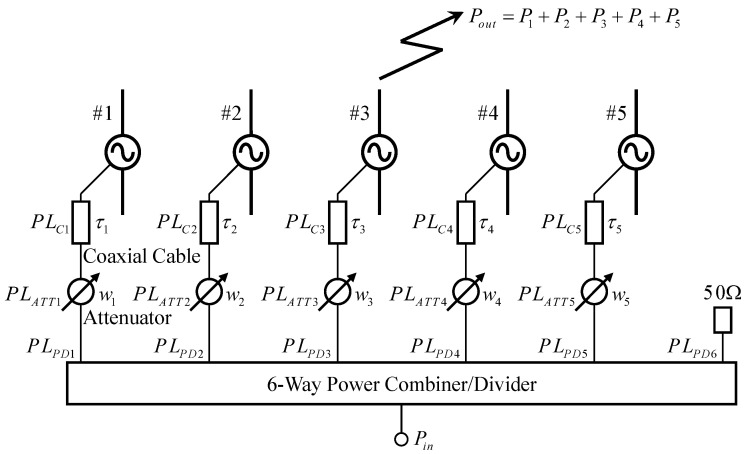
Configuration of a beamforming network.

Figure 15 illustrates the configuration of the beamforming network. The unequal power combiner, which is needed to embody the weight functions, is substituted with the available equal power combiner and attenuators. Furthermore, the phase shift is achieved by using differing lengths of fabricated coaxial cables that connect with the antenna. To the best of our knowledge, a five-way equal power combiner has not been commercially available so far. Thus, we used a six-way equal power combiner/divider (mini circuit, ZN6PD-63W-S+) for the experiments. Applying this results in a power loss due to one wasted port, which is loaded at 50 Ω. In addition, the attenuators required to make non-uniform weighting also generate serious power losses. To evaluate the beam of the proposed antenna, the total input power of the antennas must be calibrated while considering the power losses in the power combiner/divider, attenuators, and coaxial cables. Otherwise, the proposed antenna performance would be underestimated compared to the analytical outcomes. In our previous study, a calibration method to achieve Gaussian angular power spectra in a three-dimensional OTA apparatus was proposed [36]. The calibration of the beamforming for the proposed antenna is an extension of that method.

The principle of the calibration method is explained while considering it to be a transmitting antenna based on the reciprocal theorem. To obtain the reference gain for the directivity measurement, the average received power $E\left[|S_{21}{|}^{2}\right]$ of the half-wavelength dipole antenna in the full-azimuth angle was measured in an anechoic chamber. Then, the reference level *REF* is calculated using $E\left[|S_{21}{|}^{2}\right]$ based on the following equation:

(11) $$REF=\frac{E\left[|S_{21}{|}^{2}\right]}{G}$$

where *G* is the ratio of the input power *P_in_* of the beamforming network to the output power *P_out_* of the proposed antenna. The output power of the proposed antenna constructed by the five dipole antennas is calculated as follows:

(12) $$P_{out}=P_{1}+P_{2}+P_{3}+P_{4}+P_{5}$$

where *P_i_* indicates the output power of each element and is calculated, considering the power loss of each component, by the following equation:

(13) $$P_{i}=P_{in}PL_{PDi}PL_{ATTi}PL_{Ci}$$

where *PL_PDi_* denotes the ratio of the input to the output power of the equal power combiner/divider, which value is ideally one-sixth. However, the measured value *PL_PDi_* is small due to the power losses that occur in the device. *PL_ATTi_* is the insertion loss of the attenuator, whereas *PL_Ci_* is the power loss of the coaxial cable.

The attenuator is utilized to represent the weight *w_i_* mentioned in Section 2.3. The weight is defined by the voltage, which is converted to power-based value by squaring. Thereby, the attenuator of each element can be set as listed in Table 2. It should be noted that the attenuator is set up in 0.5 dB increments, considering that it has negligible effect on the beam to be formed. The setting of the attenuators is applied to the directivity measurements in the anechoic chamber, and the propagation experiments in an indoor situation are described in Section 5.

**Figure 16:**
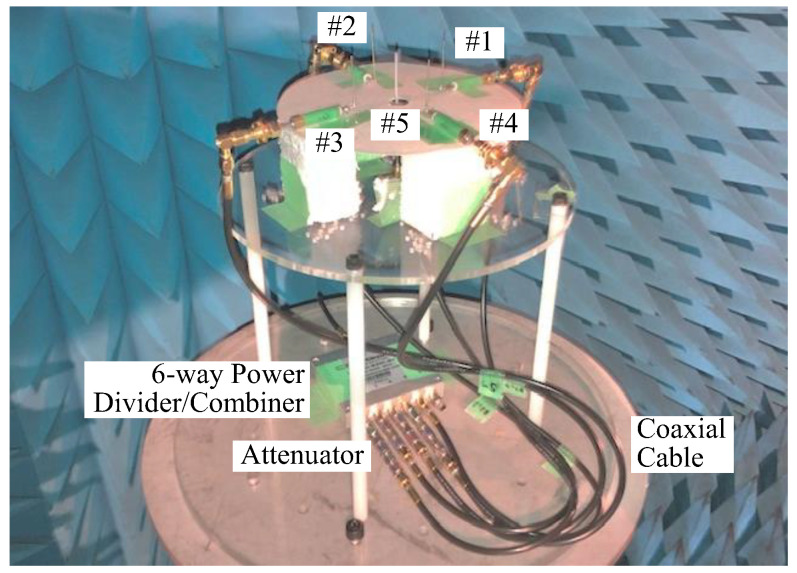
Fabricated antenna with a beamforming network.

Figure 16 shows a photograph of the fabricated antenna with the beamforming network described in Figure 15. Four dipole antennas (#1–#4), which are mounted on a circle with a radius *a*, are employed in the form of a spertopf dipole antenna, which is easy to adjust in terms of the element length of the antenna. However, we cannot locate it at the center of the circle because it has a structure with a spertopf balun. Therefore, another dipole antenna (#5) is used as a sleeve dipole antenna. The length of the dipole antenna is 6.8 cm (0.45 *λ*).

**Figure 17:**
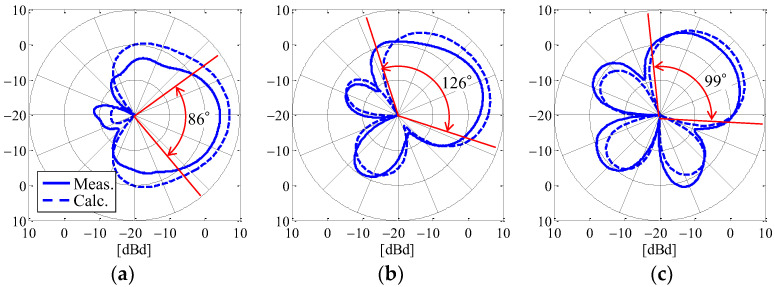
Measurement results for the radiation beam depending on the desired angle: (**a**) *ϕ* = 0°; (**b**) *ϕ* = 22.5°; and (**c**) *ϕ* = 45°.

Figure 17 shows the results of applying the calibration method to the measured directivity using a beamforming network composed of the weight function according to the desired angle. Figure 17a–c indicate the cases when the desired beamforming direction is set to 0°, 22.5°, and 45°, respectively. The solid and broken curves are the measured result and the calculated outcome, respectively. The red arrows are the full width at half maximum of the measured radiation gain.

It can be seen from Figure 17 that the beam direction changes depending on the desired beamforming direction, so the weight function works to control the beamforming. The measured results show a radiation pattern that is a little smaller than the calculated outcomes, but the shapes are almost identical. As can be seen from Figure 16, the cause of this radiation gain reduction is expected to a significant factor: the phase variation due to the bending of the coaxial cable between the attenuator and the antenna, which creates the error between the desired phase shift value and the phase of the cable. In addition, the full width at half maximum is somewhat wider than the analytical results illustrated in Figure 11, indicating that the approach is very suitable for beam steering.

By determining the optimal weight function according to the estimated angle of the incoming wave, the beam can be continuously steered in the direction of the sensor device. Since the estimated angle of the incoming wave has an error, it is unrealistic to continuously change the weight functions. Taking advantage of the large half-width of the beamforming in the proposed antenna shown in Figure 17, we investigated the number of beam steering divisions in multiple areas at the full-azimuth angle to absorb the estimation error of the angle of the incoming wave.

**Figure 18:**
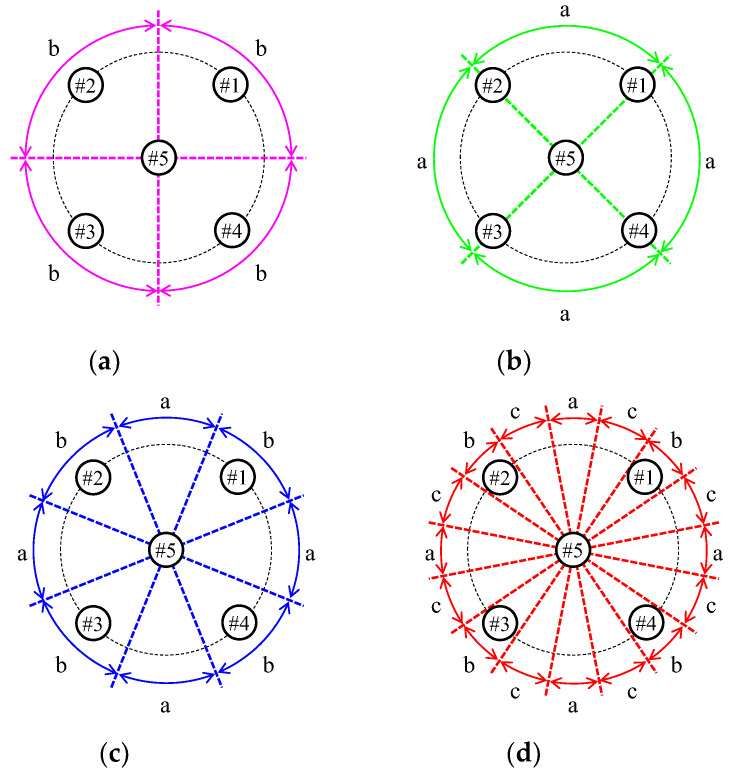
Division patterns of the beamforming area: (**a**) 90° intervals from 0°; (**b**) 90° intervals from 45°; (**c**) 45° intervals; and (**d**) 22.5° intervals.

Figure 18 draws four division patterns of the beamforming area in the full-azimuth angle. The patterns for the four divisions are shown in Figure 18a,b. The beamforming area shown in Figure 18a indicates 90° intervals from 0°. In this case, the beam is best oriented at 45°, which is the center of the division region, so only the directivity shown in Figure 17b is used. Note that the characters (a, b, and c) in Figure 18 are associated with the directivity of Figure 17. Since the proposed antenna has a symmetrical structure, the same weight function can be applied by changing the elements connected to the beamforming network even if the beam direction changes in 90° intervals. In the same way, the beamforming area shown in Figure 18b has 90° intervals from 45°, and only the directivity shown in Figure 17a is used. Two patterns with finer divisions in all directions were considered. The patterns for the cases of 8 and 16 divisions are shown in Figure 18c,d, respectively. Figure 18c utilizes the two beams shown in Figure 17a,b, whereas Figure 18c employs the three beams shown in Figure 17.

**Figure 19:**
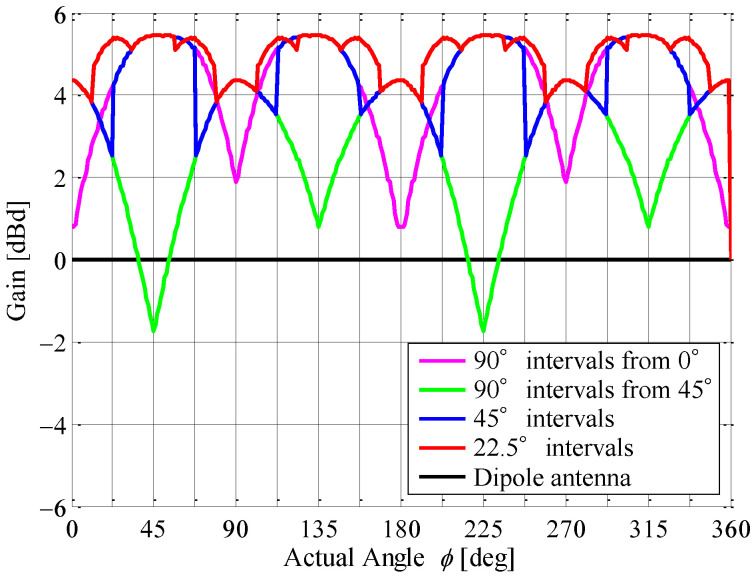
Radiation gain with respect to the angle of the incoming wave.

Figure 19 shows the radiation gain for the angle of the incoming wave by combining the measured directivities shown in Figure 17. As shown in Figure 19, the radiation gain increases with increasing number of division areas. In particular, a gain of 4 dBd or more can be maintained when all directions are divided into 22.5° intervals. These results reveal that the proposed antenna system can provide a high receiving power utilizing a beam steering function with a full-azimuth angle when compared to a conventional dipole antenna.

## 4. AOA Estimation Using a Fading Emulator

The smart gateway for healthcare networks is designed for use in indoor environments, such as a house or hospital. In such an environment, various objects in the surrounding area, such as walls, floors, ceilings, and furniture, result in a complex radio propagation environment. Furthermore, if a sensor device mounted on a person exists in the same room as the gateway, a direct wave would arrive to the gateway, which is called the Rice channel propagation environment. To verify the accuracy of the AOA estimation of the proposed antenna in a multiple-wave propagation environment, OTA testing using a two-dimensional fading emulator was executed.

A spatial fading emulator is implemented using the Monte Carlo simulation [31]. In the fading emulator, the radio waves radiated from the scatterers, which are the controlled amplitude and phases of the signal used to emulate a Rayleigh fading channel, are summed around the evaluating antennas, and the desired radio environment is then generated [37,38]. Furthermore, when the signal radiated from the extra antenna is controlled according to the features of the direct wave, such as power and direction, a Rice propagation environment can be represented [39].

**Figure 20:**
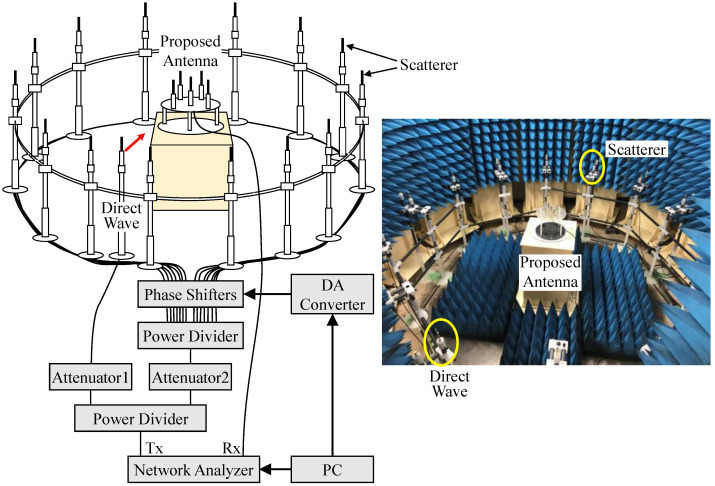
Two-dimensional fading emulator to realize a Rice propagation environment.

Figure 20 draws the measurement setup of the two-dimensional fading emulator to actualize the Rice propagation environment that can create the desired incoming wave direction and K-factor. To reproduce a Rice propagation environment in the fading emulator, 14 half-wavelength sleeve dipole antennas are arranged at equal angular intervals on a circle with a radius of 1.2 m to radiate scattered waves, while the remaining one half-wavelength sleeve dipole antenna emits a direct wave. Then, the desired K-factor, which represents actual indoor propagation environments, is achieved by controlling the output power of the direct wave or scattered waves using the method described below.

Firstly, a multiple-wave radio propagation environment with only scattered waves is implemented, and the average received power *P_r_* of a dipole antenna placed in the center of the fading emulator is measured. Next, the received power *P_d_* of a dipole antenna is measured when only a direct wave is emitted. The difference between the two received powers (*P_d_* and *P_r_*) and the K-factor are compared. The attenuators (Attenuator1 and Attenuator2 shown in Figure 20) are set using the following equation:

(14) $$\begin{array}{l} \mathrm{If}\;P_{d}\left[\mathrm{dBm}\right]-P_{r}\left[\mathrm{dBm}\right]>K\left[\mathrm{dB}\right]\\ \;\mathrm{Attenuator}1=\left(P_{d}-P_{r}\right)-K\left[\mathrm{dB}\right]\\ \;\mathrm{Attenuator}2=0\\ \mathrm{If}\;P_{d}\left[\mathrm{dBm}\right]-P_{r}\left[\mathrm{dBm}\right]<K\left[\mathrm{dB}\right]\\ \;\mathrm{Attenuator}1=0\\ \;\mathrm{Attenuator}2=K-\left(P_{d}-P_{r}\right)\left[\mathrm{dB}\right] \end{array}$$

Here, Attenuator1 is connected to a sleeve dipole antenna that emulates a direct wave, and Attenuator2 is plugged in the multi-way power divider, which generates multiple scattered waves.

**Figure 21:**
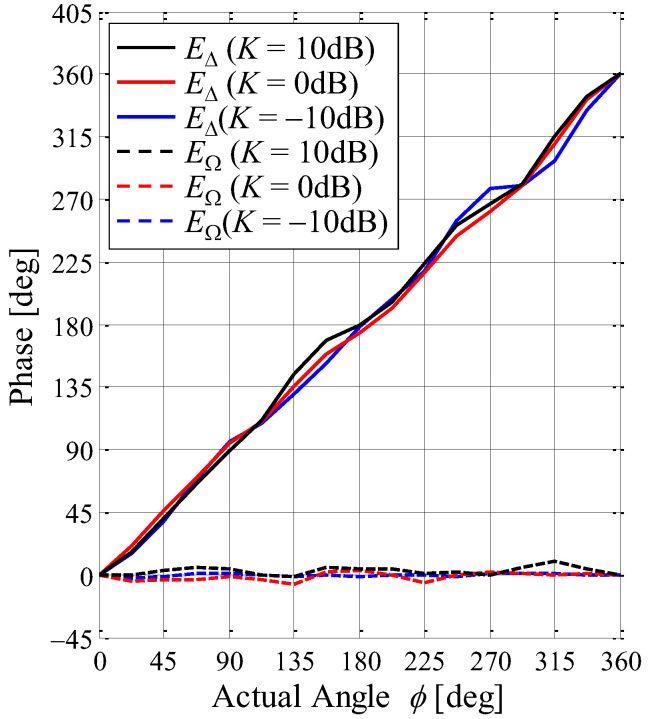
Phase characteristics vs. angle of the incoming wave.

To assess estimation accuracy, the proposed antenna is set to the center of the fading emulator, and the received signals of *E*_∆_ and *E*_Ω_ are measured using a vector network analyzer. Figure 21 shows the phase of each signal by applying the MIQ method as a function of the angle of the incoming wave with the K-factor as a parameter through the fading emulator. The black lines, red lines, and blue lines show the case of *K* = 10 dB, 0 dB, and −10 dB, respectively. The solid and broken lines indicate *E*_∆_ and *E*_Ω_, respectively.

As shown in Figure 21, there is no phase variation regardless of the K-factor because the effect of the scattered waves on the received signal is canceled by the MIQ method, as expressed in Figure 8. ∠*E*_Ω_ used as the reference angle is constant regardless of the angle of the incoming wave, and ∠*E*_∆_ varies proportionally, depending on the angle of the incoming wave.

**Figure 22:**
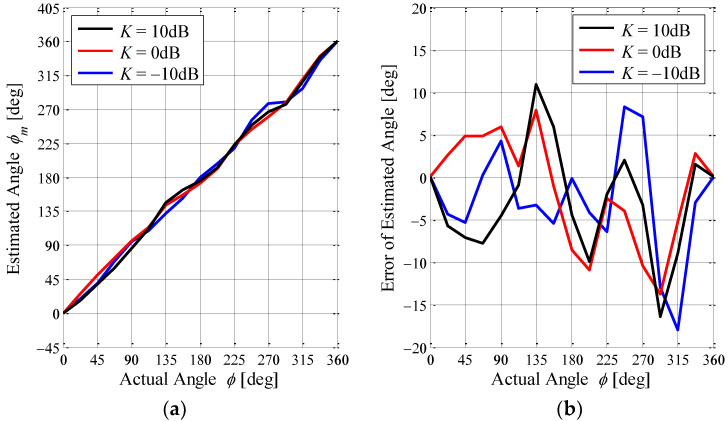
Estimation accuracy of the proposed antenna obtained through the OTA testing: (**a**) estimated angle and (**b**) error of the estimated angle.

Figure 22 shows the estimation accuracy of the proposed antenna as a function of the angle of the incoming wave with the K-factor as a parameter. Figure 22a indicates the estimated angle when applying Equation (5) to the phase of Figure 21, whereas Figure 22b is the difference between the estimated angle and the actual angle, i.e., the estimation error. The black lines, red lines, and blue lines show the case of *K* = 10 dB, 0 dB, and −10 dB, respectively.

It can be seen from Figure 22a that the estimated angle coincides with ∠*E*_∆_ because the reference angle ∠*E*_Ω_ is constant regardless of the angle of the incoming wave, as shown in Figure 21. In addition, we can observe that the estimated angle agrees with the actual angle regardless of the K-factor. In Figure 22b, an estimation error within ±15° is found regardless of the K-factor, with its features being similar to the analytical outcomes shown in Figure 10b. It is concluded from these figures that the AOA estimation using the MIQ method achieves sufficient accuracy for a small number of elements, even though the K-factor is very small. Hence, the ability of the AOA estimation for the proposed antenna, when operating in an indoor situation, can usefully form a beam with respect to the angle of the incoming wave.

## 5. Radio Propagation Experiments in an LOS Environment

To examine the effectiveness of the beam steering function in the proposed antenna, this section presents the radio propagation experiments conducted to evaluate the receiving level performance according to beam steering, which is dependent on the movement of a human who is attached to the sensor device. In this indoor situation, we performed two types of measurements: one with K-factor estimation in an LOS propagation environment with short-term fading, and one with received level characteristics from long-term fading.

**Figure 23:**
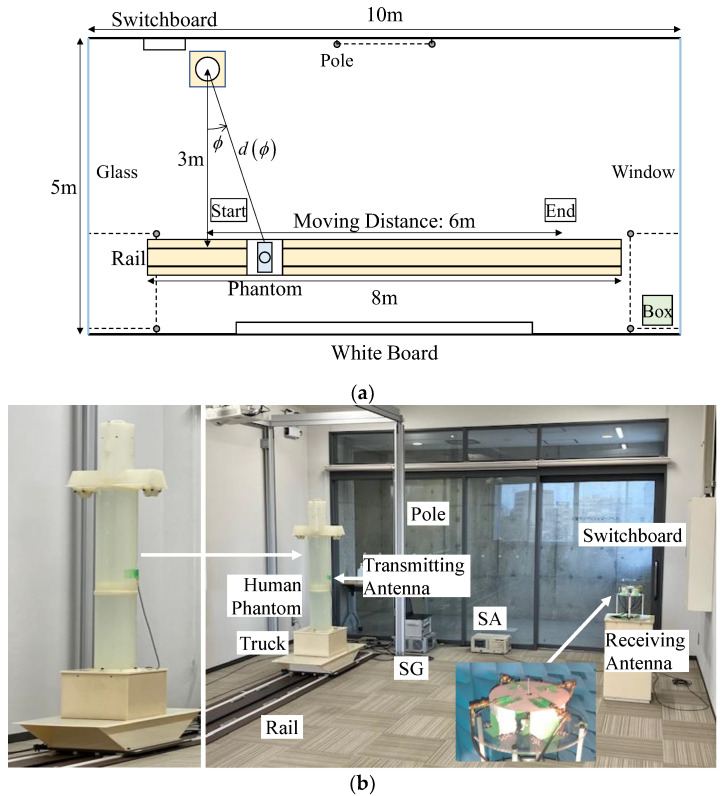
Radio propagation experiment performed in an indoor situation: (**a**) top view of the indoor measurement environment and (**b**) measurement setup in the classroom.

Figure 23a shows the top view of the indoor measurement environment, which is a 10 m × 5 m classroom at the University of Toyama. The distance from the floor to the ceiling is 3 m. A study involving humans requires us to obtain an informed consent statement from the participants, and moreover, the repeatability of measurement with real humans tends to be poor. As an alternative, we previously developed an arm-swinging dynamic phantom that can simulate the natural walking style of humans [40]. This phantom was created to be a realistic geometrical representation of an average Japanese male [41]. The head and body are approximated by circular cylinders, the dimensions of which are 18 cm in diameter by 25 cm in height, and 22 cm in diameter by 135.5 cm in height, respectively. The circular cylinders contain a mixed solution that simulates the electrical properties of a human [42].

With regard to a sensor device mounted on a human being, dynamic channel variations caused by arm swinging while walking must be considered [43]. The shadowing effects are attributed to the sensor device attached to the person and should not be included in the evaluation of the gateway system. However, variation in the direction of the incoming wave due to human movement is very important. Therefore, a half-wavelength sleeve dipole antenna, which represents a sensor device, was attached to the waist of a human phantom without arms. Moreover, the human phantom was placed on a truck and was moved at a constant speed on rails. The gap between the dipole antenna and the surface of the body was set to 1 cm. The height from the floor to the sensor device and the gateway antenna was set to 1 m. The distance from the human phantom to the wall was 1.5 m, while the distance from the proposed antenna to the wall was 0.5 m.

Figure 23b depicts a photograph of the measurement setup in a classroom. The output signal of the signal generator (SG) is radiated from the half-wavelength sleeve dipole antenna attached to the human phantom, and the received signal of the proposed antenna is measured by a spectrum analyzer (SA). The transmit signal is set to continuous wave, and the measured frequency is 2 GHz.

To assess the K-factor in the indoor situation, we conducted an experiment using the human phantom. To achieve short-term fading in off-body communication, the travel distance of the phantom was set to 0.9 m (6 *λ*) from the start point. The maximum difference in the free-space path loss on the moving distance was 0.4 dB, indicating the propagation loss variation was very small.

**Figure 24:**
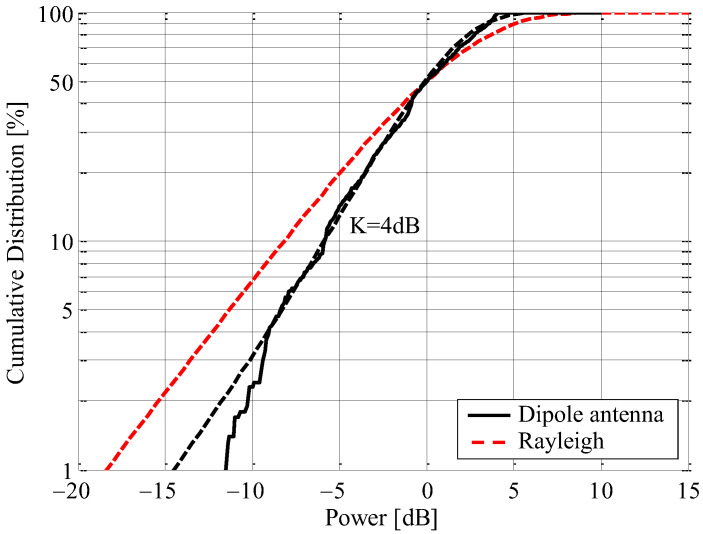
CDF characteristics of the received power in short-term fading.

The receiving antenna used is a half-wavelength dipole antenna, which is omnidirectional on the horizontal plane, to evaluate incoming wave characteristics. Figure 24 shows the cumulative distribution function (CDF) characteristics of the measured instantaneous response. The received power is normalized by its median value. The black solid curve is that of measured results. The black broken curve is the theoretical CDF characteristic for the K-factor estimated from the measured data. The theoretical curve for Rayleigh response is also included as a red broken line in the Figure.

In Figure 24, the estimated K-factor is 4 dB, despite the short-distance communication. As can be seen from Figure 23b, the cause of this characteristic appears to be reflected waves from not only the walls, ceilings, and floors, but also from an aluminum pole close to the transmitting antenna and a metal switchboard near the receiving antenna, indicating that slightly stronger fading has been observed. This fading phenomenon also occurred in the long-distance propagation experiment. Although we did not perform the AOA estimation using the proposed antenna in this room, it is expected that the angle of the incoming wave can be estimated in this environment (*K* = 4 dB), as described in Section 4.

**Figure 25:**
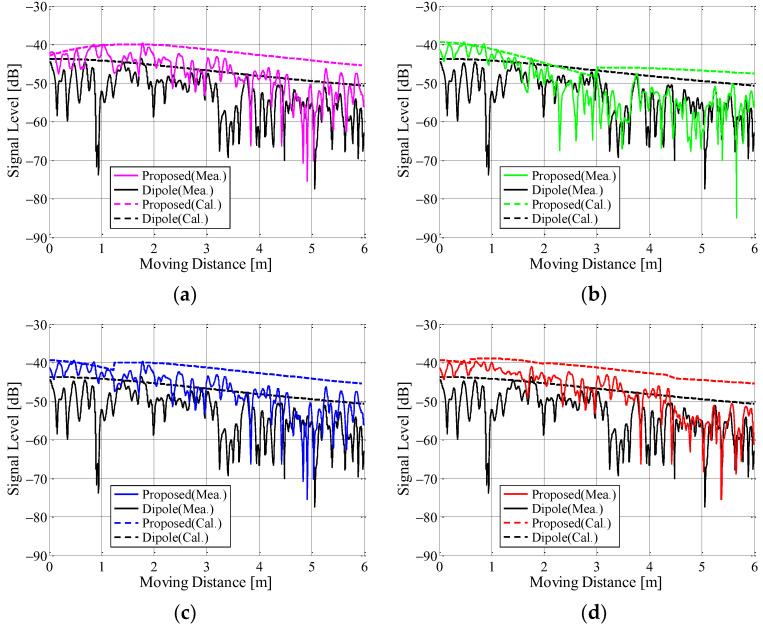
Instantaneous received signals of the proposed antenna: (**a**) 90° intervals from 0°; (**b**) 90° intervals from 45°; (**c**) 45° intervals; and (**d**) 22.5° intervals.

Figure 25 shows the measurement results of the instantaneous response with respect to the antenna input terminal power with a division pattern of the beamforming area shown in Figure 18 as the parameter. Figure 18a,b indicate the case of 90° intervals from 0° and the case of 90° intervals from 45°, respectively. Figure 18c,d are the case of 45° intervals and the case of 22.5° intervals, respectively. The received power in Figure 25 is a combination of the results measured by forming each beam shown in Figure 17 on a personal computer (PC). In addition, the black lines are the results of using a half-wavelength sleeve dipole antenna as the receiving antenna.

The broken lines are the expected results when measured in free space and are calculated using the following equation:

(15) $$P\left(\phi \right)=G_{t}\left(\phi \right)G_{r}\left(\phi \right){\left(\frac{\lambda }{4\pi d\left(\phi \right)}\right)}^{2}$$

where *G_t_*(*ϕ*) and *G_r_*(*ϕ*) indicate the radiation gain at *ϕ* of the transmitting and receiving antenna, respectively. *ϕ* is the angle of the direct wave when the direction of the starting point is defined as 0°, as shown in Figure 23a, and its values vary with the movement of the arm-swinging dynamic human phantom. *d*(*ϕ*) is the distance between the transmitting and receiving antennas. When the human phantom reaches an end point, the angle *ϕ* is 63.4°. The broken curves for the proposed antenna, excluding Figure 25a, are not smooth because of beam switching, as illustrated in Figure 18.

It can be seen from Figure 25 that the experimental results in the classroom are reduced by the fading effect compared to the expected values in free space, as shown by the broken curves in the Figure. In addition, as the phantom moves, the receiving signal level decreases because the path loss increases with increasing distance between the transmitting and receiving antennas. Despite this, the received signals for all the division patterns are increased by forming the beam toward the direction of the human phantom, when compared to the results for the dipole antenna. Comparing the four measured results using different division patterns, we can see that the result for the case of 90° intervals from 45° shown in Figure 25b is slightly lower than the others.

**Figure 26:**
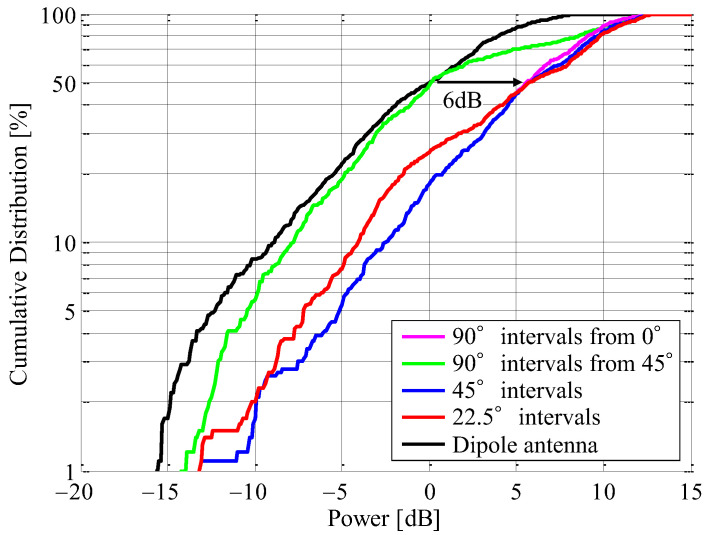
CDF characteristics of received power of each division pattern.

The CDF characteristics were analyzed to perform a statistical validation of the propagation measurement results obtained in the classroom. Figure 26 shows the CDF characteristics of the instantaneous response with the beamforming division area as the parameter. The received power is normalized by the median value measured by the dipole antenna. The pink and green curves indicate the case of 90° intervals from 0° and the case of 90° intervals from 45°, respectively. The blue and red curves are the case of 45° intervals and the case of 22.5° intervals, respectively. The black curve is the dipole antenna.

It can be seen from Figure 26 that the median value in the case of 90° intervals from 45° is the same as that of the dipole antenna. However, the CDF curve in the case of 90° intervals from 45° is on the right-hand side from that of the dipole antenna, so the received level of the proposed antenna is improved. Nevertheless, it is on the left-hand side from that of the other division patterns. This is because the directivity of the beam directed at 0°, as illustrated in Figure 17a, is smaller than the others. Therefore, the expected result in free space in the case of 90° intervals from 45° is smaller than that of the dipole antenna in the 2.5 to 3 m moving distance section.

On the other hand, in the proposed antenna, the CDF curves for the other division patterns, except for the case of 90° intervals from 45°, are improved by about 6 dB compared to those of the dipole antenna. Especially in the case of 45° intervals and the case of 22.5° intervals, the expected results in free space, as illustrated by the broken curves in Figure 25 and the CDF curves shown in Figure 26, reveal great effectiveness. Considering the simplicity of the beam steering system, it is reasonable to form beams at 45° intervals. It is concluded from these results that the introduction of the proposed antenna can either realize energy saving by reducing the radiated power at the sensor device or expand the coverage area of the proposed smart gateway.

## 6. Conclusions

This paper presents a smart gateway system that includes an AOA estimation and a beam steering function in a small circular antenna array. The fabricated antenna was assessed for its estimation accuracy of incoming wave using OTA testing in a Rice propagation environment. An estimation error within ±15° is found regardless of the K-factor, indicating that the AOA estimation using the MIQ method achieves sufficient accuracy for a small number of elements, even though the K-factor is very small. Moreover, the ability of the beam steering in the proposed antenna was evaluated through propagation measurements using a human phantom in an indoor environment. The CDF characteristic in the case when all directions are divided into 45° intervals is improved by about 6 dB compared to that of the conventional dipole antenna, indicating great effectiveness. The proposed antenna can either realize energy saving by reducing the radiated power at the sensor device or expand the coverage area of the proposed smart gateway. These outcomes show that the proposed antenna has great potential as a smart gateway system that can provide a high estimation accuracy and an enhanced received signal.

Future work may include investigations of low-profile construction of the proposed antenna for more practical use, like that used for the ESPER antenna. We have previously proposed a disk-loaded monopole stacked with a patch antenna, which is capable of radiating in all three orthogonal polarizations [44]. By using this antenna, we expect to achieve both a low profile for the gateway antenna and the reception of vertical and horizontal polarizations at the same time.

## Figures and Tables

**Table 1:** Optimal weight function.

	*ϕ* = 0°		*ϕ* = 22.5°		*ϕ* = 45°	
	*w_i_*	*τ_i_* [°]	*w_i_*	*τ_i_* [°]	*w_i_*	*τ_i_* [°]
#1	0.17	−195	0.37	−175	1.00	0
#2	0.42	−275	0.37	−300	0.62	−235
#3	0.42	−275	0.50	−255	0.54	−85
#4	0.17	−195	0.06	−310	0.62	−235
#5	1.00	0	1.00	0	0.62	−235

**Table 2:** Setup of the attenuators.

	*ϕ* = 0°			*ϕ* = 22.5°			*ϕ* = 45°		
	*w*	*w* ^2^	Att. [dB]	*w*	*w* ^2^	Att. [dB]	*w*	*w* ^2^	Att. [dB]
#1	0.17	0.03	15.5	0.37	0.14	8.5	1.00	1.00	0.0
#2	0.42	0.18	7.5	0.37	0.14	8.5	0.62	0.38	4.0
#3	0.42	0.18	7.5	0.50	0.25	6.0	0.54	0.29	5.5
#4	0.17	0.03	15.5	0.06	0.004	24.5	0.62	0.38	4.0
#5	1.00	1.00	0.0	1.00	1.00	0.0	0.62	0.38	4.0

## Data Availability

Not applicable.
